# Post-Stroke Cognitive Impairment in Georgia: Diagnostic Utility of Georgian NINDS-CSN Neuropsychological Battery

**DOI:** 10.3390/neurolint18100181

**Published:** 2026-09-23

**Authors:** Maia Tighashvili, Nina Mikeladze, Nazibrola Botchorishvili, Ann Dzagnidze, Nino Chitauri, Tamar Sakvarelidze, Marina Janelidze, Zurab Nadareishvili

**Affiliations:** 1 Department of Clinical Neurology, Tbilisi State Medical University, 33 Vaja-Pshavela Avenue, 0159 Tbilisi, Georgia; nbphosta@gmail.com (N.B.); dzagnidze@hotmail.com (A.D.); ta.sakvarelidze@gmail.com (T.S.); marjaneli@yahoo.com (M.J.); 2 Department of Neurology, S. Khechinashvili University Hospital, 33 Chavchavadze Avenue, 0179 Tbilisi, Georgia; ninamikeladze@hotmail.com (N.M.); chitauri_nino@mail.ru (N.C.); 3 Stroke Center, VHC Health, 1715 North George Mason Drive, Suite #107, Arlington, VA 22205, USA

**Keywords:** post-stroke cognitive impairment, vascular cognitive impairment, ischemic stroke, NINDS-CSN, neuropsychological assessment, Georgia

## Abstract

**Background**: Post-stroke cognitive impairment (PSCI) affects approximately half of stroke survivors within the first year. Data from low- and middle-income countries, including Georgia, are limited. Although a Georgian-language version of the Montreal Cognitive Assessment (MoCA) has previously been validated as a brief cognitive screening instrument, no validated Georgian-language multidomain neuropsychological battery for vascular or post-stroke cognitive impairment is currently available. This study aimed to validate the Georgian version of the National Institute of Neurological Disorders and Stroke–Canadian Stroke Network (NINDS–CSN) neuropsychological battery by assessing its concurrent validity with the Montreal Cognitive Assessment (MoCA), and to evaluate its clinical utility for estimating PSCI prevalence. **Methods**: Patients with ischemic stroke attending a 6-month follow-up visit were prospectively enrolled. The control group (CG) comprised healthy adults. The Georgian NINDS–CSN 30 min battery was administered. Validity for diagnosing PSCI was assessed using receiver operating characteristic (ROC) curve analysis, with area under the curve (AUC) as the primary measure. **Results**: A total of 175 participants were included (73 stroke patients and 102 healthy controls); 34 of 73 stroke patients (46.6%) met the clinical reference criteria for PSCI. In demographically adjusted analyses (*n* = 72), the four-test NINDS-CSN composite demonstrated good discrimination for clinically defined PSCI (AUC, 0.821); the optimal threshold of −1.144 yielded 78.8% sensitivity and 82.1% specificity. Incorporation of TMT-A and TMT-B into a six-test composite modestly increased the AUC to 0.861, with an optimal threshold of −1.728, 72.7% sensitivity, and 94.9% specificity. The MoCA demonstrated higher discriminative performance (AUC, 0.978; 95% CI, 0.952–1.000); at the previously validated cutoff of ≤22, sensitivity was 97.0%, and specificity was 79.5%. The MoCA AUC was significantly higher than that of the six-test NINDS-CSN composite (AUC difference, 0.117; 95% CI, 0.031–0.204; *p* = 0.008). **Conclusions**: The Georgian NINDS–CSN battery demonstrated good discriminative performance. However, given the superior performance of the Georgian MoCA, it may be preferred for screening PSCI in routine practice, while the NINDS–CSN battery remains suitable for comprehensive cognitive assessment.

## 1. Introduction

Vascular cognitive impairment (VCI) is the second most common cause of cognitive impairment after Alzheimer’s disease, and post-stroke cognitive impairment (PSCI) represents a major contributor to VCI in high-income countries [1,2,3]. However, relatively few studies have examined PSCI and post-stroke dementia in low- and middle-income countries. In hospital-based samples, the reported prevalence of PSCI varies widely: 12% in Mexico, 13.6% in Ghana, 20% in China, and up to 39.9% in Nigeria [4,5,6,7]. These differences are most likely explained by variations in study methodology and the timing of PSCI assessment. A large individual-participant-data meta-analysis from the Stroke and Cognition Consortium (STROKOG) further demonstrated the substantial burden of post-stroke cognitive impairment across diverse populations, while highlighting variability related to demographic, clinical, and methodological factors [8].

In a previous population-based study, we reported a 13.4% prevalence of mild cognitive impairment in Georgia [9]. However, data on the epidemiology of PSCI in Georgia are lacking. Conducting robust epidemiological and clinical studies of PSCI requires validated cognitive assessment tools in the Georgian language. A Georgian-language version of the Montreal Cognitive Assessment (MoCA) has previously been validated as a brief cognitive screening instrument [10]. However, no validated Georgian-language multidomain neuropsychological battery specifically suitable for vascular or post-stroke cognitive assessment is currently available. We therefore sought to evaluate a Georgian version of the National Institute of Neurological Disorders and Stroke–Canadian Stroke Network (NINDS-CSN) neuropsychological battery, which provides assessment across multiple cognitive domains and has been recommended within vascular cognitive impairment harmonization frameworks [2,11,12].

Previous comparative studies have suggested that brief global screening and NINDS-CSN protocols may differ in diagnostic performance after stroke. In particular, Dong et al. reported superior discrimination of vascular cognitive impairment with the MoCA compared with the NINDS-CSN 5 min protocol, although direct comparisons involving the more extensive 30 min NINDS-CSN protocol remain limited [13].

More recently, the VasCog-2-WSO criteria have updated the diagnostic framework for vascular cognitive impairment and dementia, emphasizing objective cognitive impairment, the distinction between mild and major cognitive disorder, and the attribution of functional dependence specifically to cognitive deficits [14]. In parallel, recent international consensus recommendations have updated the approach to neuropsychological assessment in vascular cognitive impairment and dementia, emphasizing harmonized multidomain evaluation and building on the original NINDS-CSN harmonization standards [15].

The aim of this study was to evaluate the diagnostic performance of the Georgian NINDS-CSN neuropsychological battery for identifying clinically defined PSCI and to compare its observed discriminative performance with that of the previously validated Georgian MoCA.

## 2. Methods

### 2.1. Patients and Controls

This study was conducted at S. Khechinashvili University Hospital. The study protocol and informed consent form were approved by the local ethics committee. This was a prospective observational study with a control group. Patients with ischemic stroke were prospectively enrolled at the time of outpatient follow-up, and a control group (CG) of healthy participants was recruited to establish normative values.

Patients with ischemic stroke were recruited and prospectively enrolled from a cohort of outpatients who had been discharged from the hospital between January 2020 and January 2022 and subsequently attended the outpatient neurology clinic at S. Khechinashvili University Hospital. Participant recruitment and inclusion in the final analytic sample are summarized in Figure 1.

Patient visits included in-person neurologic examination. An experienced board-certified neurologist performed the clinical reference assessment before administration of the study index tests. The assessment included the history and temporal evolution of cognitive symptoms, patient- and/or informant-reported decline from the premorbid level of cognitive functioning, functional status, neurological examination, and review of relevant laboratory and neuroimaging findings. The diagnostic framework was based on the VASCOG criteria for vascular cognitive disorders [16]. For the purposes of this study, the clinical reference diagnosis of post-stroke cognitive impairment (PSCI) required: (a) evidence of an acquired decline from the previous level of cognitive functioning together with (b) objective evidence of cognitive impairment. Objective evidence of cognitive impairment within the clinical reference assessment was based on the Mini-Mental State Examination (MMSE; PAR, Inc., Lutz, FL, USA), with a score < 26 used as the operational threshold. A temporal relationship of ≤6 months between the index stroke and the onset of cognitive impairment was required to establish the diagnosis of PSCI [2]. Functional limitations attributable primarily to motor, sensory, or other physical consequences of stroke were not considered evidence of cognitive functional decline. Final clinical diagnosis of PSCI was based on a combination of above-described criteria including patient/informant interview, neurological exam, and MMSE. At the conclusion of the clinical appointment, the patient was invited to participate in a psychometric study on the same day. Those interested provided consent and underwent testing on the MoCA (MoCA Test Inc., Greenfield Park, QC, Canada) and NINDS-CSN battery by an independent rater who was not informed of the clinical diagnosis. All participants were Georgian-speaking and were aged 50 years and older. Age, sex, ethnicity, and years of education were recorded for all subjects, including patients and controls.

Patients with ischemic stroke aged ≥50 years, without preexisting medical or psychiatric conditions affecting cognitive function, and who were fluent in the Georgian language were eligible for inclusion. Patients with documented pre-stroke dementia, intracerebral hemorrhage, or subarachnoid hemorrhage were excluded. Patients with aphasia were also excluded. Written informed consent was obtained from all participants or their caregivers (in cases of cognitive impairment), using forms approved by the local ethics committee. Patients with stroke were further stratified into two groups: those meeting criteria for PSCI and those without PSCI. PSCI was treated as a binary clinical outcome for the primary diagnostic-accuracy analyses and was not formally subclassified into mild and major vascular cognitive disorder.

A control group (CG) was recruited to define normative values for the NINDS-CSN battery. All control participants were recruited according to a predefined protocol and underwent structured screening to exclude neurological, psychiatric, or cognitive disorders. CG consisted of healthy adults recruited from a convenience sample of caregivers accompanying patients attending the outpatient clinic. Eligible individuals had no history of neurologic disorders or subjective memory concerns. All CG participants underwent screening for medical history, and those reporting neurologic disease or cognitive complaints were excluded. Additional exclusion criteria included sensory deficits limiting test performance (e.g., severe visual or hearing impairment) and medical conditions associated with cognitive impairment, such as developmental disorders, significant substance abuse, severe psychiatric illness, traumatic brain injury, or intracranial space-occupying lesions. No formal age matching was performed; however, participants aged ≥ 50 years were preferentially recruited. Normative values for the Georgian version of the neuropsychological tests were derived from this CG sample.

Demographic characteristics included age, sex, educational level, and residence (urban vs. rural). Higher education was defined as completion of a 5-year college-equivalent program.

Vascular risk factors, including smoking, alcohol use, hypertension, diabetes mellitus, hyperlipidemia, prior myocardial infarction, and atrial fibrillation, were systematically recorded. Infarct location and severe white-matter disease were assessed using CT or MRI. Stroke etiology was classified according to the TOAST criteria on the basis of neurovascular imaging and cardiac evaluation. Stroke severity was assessed using the NIH Stroke Scale (NIHSS), and functional outcome was assessed using the modified Rankin Scale (mRS).

Standardized mean differences between the stroke and control groups were quantified using Cohen’s d, calculated as the difference between group means divided by the pooled standard deviation. Absolute values of Cohen’s d are presented in the tables, with values of approximately 0.2, 0.5, and 0.8 conventionally interpreted as small, medium, and large effects, respectively.

### 2.2. Neuropsychological Testing

In accordance with recommendations from the American Heart Association/American Stroke Association (AHA/ASA) and VICCCS-2, we administered the NINDS-CSN 30 min neuropsychological battery [2,11,12]. This battery includes the Semantic Fluency Test (animal naming), the Phonemic Fluency Test (COWAT F-A-S), the Digit Symbol Substitution Test (Wechsler Adult Intelligence Scale–Third Edition; The Psychological Corporation, San Antonio, TX, USA), the Hopkins Verbal Learning Test (HVLT-R; PAR, Inc., Lutz, FL, USA), the Center for Epidemiologic Studies Depression Scale, and the Neuropsychiatric Inventory Questionnaire (NPI-Q) [11]. Consistent with VICCCS-2 and AHA/ASA recommendations, we also administered the Georgian versions of the Montreal Cognitive Assessment (MoCA) and the Trail Making Tests A and B (TMT-A and TMT-B) [2,10,12].

The NINDS-CSN neuropsychological battery was translated and adapted for use in Georgian-speaking participants before study administration. The original English-language materials were translated into Georgian by two translators. An independent bilingual translator performed back-translation into English. The translated version was reviewed by neuropsychologists for semantic equivalence, comprehensibility, and cultural appropriateness. Discrepancies were resolved by consensus. Particular attention was given to verbally mediated tasks, including the Hopkins Verbal Learning Test and phonemic verbal fluency, for which linguistic characteristics may influence performance. The resulting Georgian version was pilot-tested in 20 Georgian-speaking individuals who were not included in the final study sample, and modifications were made where necessary to improve clarity and cultural appropriateness before its use in the study.

### 2.3. Definitions

The clinical reference diagnosis of PSCI was established independently as described in Section 2.1. MoCA and NINDS-CSN classifications were considered index-test classifications and were not used to establish the clinical reference diagnosis. Based on the MoCA, cognitive impairment was defined using a cutoff score of ≤22. For the NINDS-CSN 30 min battery, cognitive impairment according to the normative criterion was defined as a z-score ≤ −1.5 on at least one of the following four core cognitive measures: Hopkins Verbal Learning Test (HVLT), phonemic verbal fluency (COWAT F-A-S), semantic verbal fluency (Animal Fluency), or Digit Symbol Substitution Test (DSST) [10,11]. This test-based classification was considered separately from the clinical reference diagnosis of PSCI. A continuous NINDS-CSN cognitive summary score was also calculated from these four measures for the diagnostic-accuracy analyses, as described below. TMT-A and TMT-B were considered supplemental measures and were not included in the primary four-test composite. Functional and neuropsychiatric measures, including ADL, IADL, CES-D, and NPI-Q, were analyzed separately and were not included in the cognitive summary score to define PSCI.

### 2.4. Statistical Analysis

Continuous variables are presented as mean ± standard deviation (SD) or median, as appropriate, and categorical variables as number and percentage. Between-group comparisons were performed using Welch’s *t*-test for approximately normally distributed continuous variables and the Mann–Whitney U test for non-normally distributed or ordinal variables. Categorical variables were compared using the Pearson chi-square test or Fisher’s exact test when expected cell counts were small. All statistical tests were two-sided, and *p* < 0.05 was considered statistically significant. Because analyses of clinical and neuroimaging characteristics were exploratory and involved multiple comparisons, no adjustment for multiple testing was applied, and these findings were interpreted cautiously.

For the primary NINDS-CSN analysis, raw scores on the four core cognitive measures—HVLT, COWAT F-A-S, Animal Fluency, and DSST—were standardized relative to the healthy-control group using the formula: z = $\frac{X-\mu \;control}{\sigma \;control}$, where X represents the participant’s raw score and μ and δ represent the mean and standard deviation of the healthy-control group for the corresponding test. For each participant, the arithmetic mean of the four component z scores was calculated to generate the continuous NINDS-CSN cognitive summary score. Higher summary scores indicate better cognitive performance. TMT-A and TMT-B were analyzed as supplemental measures and were not included in the primary four-test NINDS-CSN composite, consistent with the structure of the NINDS-CSN 30 min protocol. Because longer TMT completion times indicate poorer performance, the direction of TMT z-scores was reversed after standardization so that, for all cognitive measures, lower z-scores consistently represented poorer performance [17]. TMT-A and TMT-B were discontinued after 100 and 300 s, respectively; participants who did not complete the task correctly within these limits were assigned the prespecified administrative failure values of 101 and 301 s, respectively.

The primary normative analysis was based on the available cognitive data from the full healthy-control sample. Because neuropsychological performance may be influenced by demographic characteristics, an additional sensitivity analysis was performed using regression-based normative adjustment for age, sex, and educational level. For each cognitive measure, a linear regression model was fitted in healthy controls with the cognitive test score as the dependent variable and age, sex, and education as predictors [18]. Each patient’s demographically adjusted z-score was calculated from the difference between the observed and demographically predicted score, standardized by the residual standard deviation of the corresponding control regression model.

Receiver operating characteristic (ROC) curve analysis was used to evaluate discrimination of the continuous NINDS-CSN summary score against the clinical reference diagnosis of PSCI. Diagnostic performance was summarized by the area under the ROC curve (AUC), and the cutoff maximizing the Youden index (sensitivity + specificity − 1) was selected as the optimal threshold [19]. To examine the effect of incorporating the full set of administered NINDS-CSN measures, analyses were performed using both a four-test and a six-test composite. The four-test composite included HVLT, COWAT F-A-S, Animal Fluency, and DSST, whereas the six-test composite additionally included TMT-A and TMT-B. For each composite, the arithmetic mean of the standardized scores was calculated and evaluated using the same ROC procedure. Areas under correlated ROC curves were compared using DeLong’s method, with the difference in AUC reported together with its 95% confidence interval and *p* value [20].

No missing values were imputed. All 73 stroke participants had complete data for the four cognitive measures included in the primary NINDS-CSN composite (HVLT, COWAT F-A-S, Animal Fluency, and DSST), as well as for MoCA and MMSE; therefore, the primary cognitive and ROC analyses included the full stroke sample. For healthy-control normative calculations, test-specific means and standard deviations were calculated using all available observations for each cognitive measure. Cognitive data were available for 102 controls for HVLT and for 101 controls for COWAT F-A-S, Animal Fluency, DSST, TMT-A, and TMT-B.

Statistical analyses were performed using IBM SPSS Statistics version 22.0 (IBM Corp., Armonk, NY, USA) and jamovi version 2.6.26 (The jamovi project, Sydney, Australia).

## 3. Results

We studied 73 stroke patients and 102 control group (CG) participants. Compared with controls, stroke patients were more likely to have higher education (73.9% vs. 51.9%, *p* = 0.003) but were less likely to be female (72.6.% vs. 47.9%, *p* = 0.002) and to reside in urban areas (83.6% vs. 93.2%, *p* = 0.02) (Table 1). The median NIHSS score was 7. Overall, 60.3% of patients had an NIHSS score of 5–15 and 8.2% had an NIHSS score > 15, indicating that a substantial proportion of the cohort had moderate-to-severe neurological deficits (Table 2).

Clinical and neuropsychological assessment of patients was conducted at 6.8 ± 0.7 months after the index stroke. Compared to the CG, stroke patients showed significantly lower scores on all cognitive tests studied (Table 1). In addition, stroke patients demonstrated significantly higher depressive scores (Table 1). NPI-Q data were available only for the stroke cohort; among these patients, 5 had at least one reported neuropsychiatric symptom.

Clinical and neuroimaging characteristics according to the clinical reference diagnosis of PSCI are summarized in Table 2. Patients with clinically defined PSCI were older than those without PSCI (70.62 ± 11.36 vs. 64.69 ± 11.45 years; *p* = 0.030). Thalamic infarction was observed exclusively in the PSCI+ group (14.7% vs. 0%; *p* = 0.019), and moderate-to-severe white-matter disease was more frequent among patients with PSCI (47.1% vs. 23.1%; *p* = 0.031). Cryptogenic stroke etiology was also more frequent in the PSCI+ group (29.4% vs. 7.7%; *p* = 0.016). A left-hemisphere infarct was numerically more common among patients with PSCI, although the difference did not reach statistical significance (50.0% vs. 28.2%; *p* = 0.056). No significant between-group differences were observed in sex, educational attainment, vascular risk factors, NIHSS severity distribution, discharge mRS, or most other infarct locations and etiologic subtypes. Given the number of unadjusted comparisons and the relatively small sample size, these associations should be regarded as exploratory.

Neuropsychological and functional performance according to the clinical reference diagnosis is presented in Table 3. According to the clinical reference diagnosis, 34 of 73 patients (46.6%) met criteria for PSCI. Patients with clinically defined PSCI performed significantly worse than those without PSCI on all cognitive measures, including HVLT, COWAT F-A-S, Animal Fluency, DSST, TMT-A, TMT-B, MoCA, and MMSE (all *p* < 0.001). The magnitude of between-group differences was large for the individual cognitive measures, with Cohen’s d ranging from 0.928 for Animal Fluency to 2.307 for the MoCA. Clinically defined PSCI was also associated with poorer functional performance, reflected by lower ADL (*p* = 0.006) and IADL scores (*p* < 0.001), and with greater depressive symptom burden on the CES-D (*p* = 0.003). These effect sizes are presented descriptively and should not be interpreted as independent measures of diagnostic validity, particularly because the MMSE contributed to the clinical reference assessment.

In the primary analysis using unadjusted normative z-scores derived from the control sample, the four-test NINDS-CSN composite demonstrated good discrimination for clinically defined PSCI, with an AUC of 0.858. The Youden-optimal threshold was—1.259, yielding a sensitivity of 70.6% and a specificity of 87.2%. When TMT-A and TMT-B were incorporated into the exploratory six-test composite, the AUC was 0.870. The optimal threshold of—1.616 yielded a sensitivity of 82.4% and a specificity of 84.6%. In the sensitivity analysis using normative z-scores adjusted for age, sex, and education, the four-test composite yielded an AUC of 0.821. The corresponding optimal threshold was −1.144, with a sensitivity of 78.8% and specificity of 82.1% (Figure 2). When TMT-A and TMT-B were additionally incorporated into an exploratory six-test composite, the demographically adjusted AUC was 0.861, compared with 0.821 for the four-test composite (Figure 3). For the six-test composite, the optimal threshold was −1.728, yielding 72.7% sensitivity and 94.9% specificity. Thus, demographic adjustment did not alter overall discriminative performance, although the sensitivity–specificity balance at the internally selected optimal thresholds differed. Across both the primary and sensitivity analyses, incorporation of TMT-A and TMT-B resulted in only a modest increase in overall discriminative performance.

The AUC of the MoCA was 0.978 (95% CI, 0.952–1.000) (Figure 2). At the previously validated cutoff score of ≤22, the MoCA had a sensitivity of 97.0% and a specificity of 79.5% for detecting clinically defined PSCI. Figure 3 shows the ROC curves for the MoCA and the demographically adjusted six-test NINDS-CSN composite. The observed AUC of the MoCA was significantly higher than that of the NINDS-CSN composite (0.978 vs. 0.861; AUC difference, 0.117; 95% CI, 0.031–0.204; *p* = 0.008). Using a MoCA cutoff of ≤22, 40 of 73 patients (54.7%) were classified as cognitively impaired by the MoCA.

## 4. Discussion

In this study, we demonstrated that the Georgian version of the NINDS-CSN neuropsychological battery is feasible and shows good diagnostic utility in patients assessed, on average, approximately 6 months after stroke. To account for demographic differences between the stroke and control samples, cognitive scores were standardized using regression-based normative models adjusted for age, sex, and education. The demographically adjusted four-test cognitive summary score, comprising HVLT, COWAT F-A-S, Animal Fluency, and DSST, demonstrated good discrimination for clinically defined PSCI, with an AUC of 0.821. At the internally derived optimal threshold of −1.144, sensitivity was 78.8%, and specificity was 82.1%. When TMT-A and TMT-B were additionally incorporated into a six-test composite, the AUC increased modestly to 0.861; the optimal threshold of −1.728 yielded a sensitivity of 72.7% and specificity of 94.9%. Thus, inclusion of the TMT measures resulted in a modest improvement in overall discrimination, primarily through increased specificity, reflecting the specific executive processing and visual-motor demands evaluated by these tasks [21].

Studies evaluating the discriminatory performance of NINDS-CSN summary scores have reported broadly comparable results. In mainland Chinese stroke patients, AUCs of 0.88, 0.88, and 0.86 were reported for the 60-, 30-, and 5 min protocols, respectively [17]. Previous studies from Singapore reported corresponding AUCs of 0.90, 0.89, and 0.79, whereas a Hong Kong study reported AUCs of 0.79, 0.79, and 0.76 [22,23]. Against this background, the AUCs observed in the present study for the demographically adjusted Georgian four-test and six-test composites (0.821 and 0.861, respectively) fall within the range of discriminatory performance reported in other cultural and linguistic settings.

Using the normative criterion of an adjusted z-score ≤ 1.5 on at least one cognitive measure, 55 of 73 stroke patients (75.3%) were classified as cognitively impaired using the four-test NINDS-CSN battery. When TMT-A and TMT-B were additionally incorporated into the six-test classification, 62 of 73 patients (84.9%) met the same criterion. These proportions represent NINDS-CSN-defined cognitive impairment and should be distinguished from the prevalence of clinically diagnosed PSCI. The higher proportion identified by the six-test approach likely reflects, at least in part, the additional contribution of TMT-A and TMT-B, on which a substantial number of stroke participants demonstrated markedly impaired performance or were unable to complete the tasks within the prespecified time limits. The overall high frequency of cognitive impairment may also be related to the clinical characteristics and severity of the stroke cohort. Stroke severity is a recognized predictor of post-stroke cognitive impairment and dementia, and findings from the Oxford Vascular Study demonstrated a marked increase in the risk of post-stroke dementia with increasing NIHSS severity [24].

Although these test-defined frequencies are not directly comparable with estimates based on clinical diagnostic criteria, previous studies provide useful context for the burden of cognitive impairment after stroke. In the international STROKOG collaboration, 44% of participants demonstrated impairment in global cognition 2–6 months after stroke or TIA, whereas the Korean multicenter VCI Harmonization Standards study identified vascular cognitive impairment in 62.6% of patients 3 months after ischemic stroke [25,26]. In the French GRECOG-VASC cohort, post-stroke neurocognitive disorder was identified in 49.5% of patients assessed at 6 months [27]. Meta-analytic estimates have generally ranged from approximately 38% to 46%, although considerable heterogeneity across studies has been attributed to differences in study populations, timing of assessment, diagnostic definitions, cognitive batteries, and impairment thresholds [28,29].

Beyond cognitive impairment, stroke patients also demonstrated a greater burden of depressive symptoms, as measured by the CES-D. Post-stroke depression is a well-recognized complication of stroke; a recent meta-analysis estimated an overall prevalence of approximately 27% [30].

In the present cohort, the MoCA demonstrated excellent discrimination for clinically defined PSCI, with an AUC of 0.978; at the previously validated cutoff of ≤22, sensitivity was 97.0%, and specificity was 79.5%. Although the MoCA and NINDS-CSN were evaluated against the same clinical reference diagnosis, the two instruments serve different clinical purposes. The MoCA is a brief global cognitive screening instrument, originally developed for mild cognitive impairment rather than specifically for stroke populations [31], whereas the NINDS-CSN provides a more detailed multidomain assessment of cognitive performance. Cognitive test performance after stroke may be influenced by focal neurological deficits, including aphasia, visuospatial neglect, visual impairment, and motor impairment, independently of cognitive dysfunction [15,32]. Individual measures within the NINDS-CSN battery, particularly verbally mediated and visual-motor tasks, are not exempt from these effects. Although patients with aphasia were excluded from the present study, other stroke-related communication, visuospatial, visual, or motor deficits may still have influenced performance on both instruments. The multidomain structure of the NINDS-CSN battery may nevertheless permit more detailed interpretation of performance across individual cognitive measures than a single global screening score. Thus, their diagnostic performance should be interpreted in the context of these different roles rather than as evidence that one instrument necessarily replaces the other.

The higher observed discriminative performance of the MoCA in our cohort is not without precedent. Dong et al. similarly reported better diagnostic performance of the MoCA than the NINDS-CSN 5 min protocol after stroke [13]. However, because their study evaluated the shorter 5 min protocol, direct comparison with the longer multidomain NINDS-CSN assessment used in the present study is limited.

The apparent association between cryptogenic stroke and a higher frequency of cognitive impairment may partly reflect confounding by age and other clinical characteristics [33]. Age may be particularly relevant because it was associated with performance on some cognitive measures in our demographic analyses, most notably the DSST. Thalamic infarction was also more frequent among patients with PSCI, which is consistent with the recognized role of the thalamus in cognitive networks [34]. However, given the small number of cryptogenic and thalamic cases and the absence of multivariable adjustment, these associations should be considered exploratory.

This study has several limitations. First, the enrolled stroke cases did not constitute a consecutive series of patients admitted to S. Khechinashvili University Hospital. Recruitment and follow-up were conducted during the early phase of the COVID-19 pandemic, which substantially disrupted outpatient assessment, while remote administration of the NINDS-CSN battery was not feasible. This may have introduced selection bias and may have affected both the observed frequency of cognitive impairment and the spectrum of patients included in the diagnostic-accuracy analyses. In addition, this was a single-center study, which may limit the generalizability of the findings to other clinical settings and populations.

Second, no formal sample-size calculation was performed in advance. The sample size was determined by the number of eligible participants who could be recruited and fully assessed during the study period. The relatively modest sample therefore limits the precision of the diagnostic-accuracy estimates and the ability to detect smaller differences between the instruments. Accordingly, 95% confidence intervals are reported for the AUC estimates to convey their statistical precision.

Third, the healthy-control sample was modest and was not designed to establish definitive population-based Georgian normative values. Although cognitive-test data were available for approximately 102 controls, complete age, sex, and education data required for regression-based demographic adjustment were available for only 73 controls. Moreover, the demographic composition of the stroke and control groups differed, particularly with respect to sex distribution, making demographic adjustment important for interpretation of the normative comparisons. Cognitive scores were therefore standardized using regression-based normative models adjusted for age, sex, and education. However, the limited size of the normative sample restricted the precision and complexity of these models and precluded robust demographic stratification. One stroke participant also lacked education data and could not be included in analyses requiring complete demographic information. Larger population-based normative samples spanning broader ranges of age, educational attainment, and other relevant demographic characteristics are needed to establish more robust Georgian reference values.

Fourth, test–retest and inter-rater reliability of the Georgian NINDS-CSN battery were not assessed. Because the battery comprises measures of distinct cognitive domains rather than multiple items intended to measure a single latent construct, internal consistency was not considered an adequate substitute for these forms of reliability. Prospective studies evaluating the reproducibility of the Georgian adaptation are therefore warranted.

An additional limitation concerns the clinical reference standard. Although neither of the index tests under evaluation—the MoCA or the NINDS-CSN battery—was incorporated into the reference diagnosis, the MMSE was used as the objective cognitive measure within the clinical assessment. The reference standard was therefore not entirely independent of global cognitive screening as a construct. This may be particularly relevant to the observed performance of the MoCA, which, like the MMSE, is a global cognitive screening instrument. The comparative diagnostic-accuracy findings should therefore be interpreted with appropriate caution.

Patients with aphasia were also excluded. Although this reduced the risk that language impairment would confound performance on verbally mediated tests, it may limit the generalizability of our findings to the broader stroke population, particularly patients with clinically significant language impairment.

Finally, the present study did not formally subclassify clinically diagnosed PSCI into mild and major cognitive disorder, and the relatively modest sample size did not permit robust multivariable evaluation of clinical factors associated with cognitive impairment. Future studies incorporating contemporary diagnostic frameworks, larger normative samples, and independent multidomain reference assessments will be important for further validation of the Georgian NINDS-CSN battery.

In conclusion, the Georgian version of the NINDS-CSN neuropsychological battery was feasible to administer and demonstrated good discriminative performance for clinically defined PSCI. Demographically adjusted four-test and six-test composite scores showed good diagnostic discrimination, with modestly higher overall performance after incorporation of TMT-A and TMT-B. The Georgian MoCA showed higher observed discriminative performance in this cohort and remains well suited to brief cognitive screening, whereas the NINDS-CSN battery provides more detailed assessment across multiple cognitive domains. These instruments should therefore be viewed as potentially complementary rather than interchangeable. Given the modest sample size, limited normative sample, single-center design, and potential construct-level dependency of the clinical reference standard arising from use of the MMSE, the comparative diagnostic findings should be interpreted cautiously. Larger multicenter studies using independent reference standards and demographically robust normative data are needed to confirm the diagnostic utility of the Georgian NINDS-CSN battery and further define its role in post-stroke cognitive assessment.

## Figures and Tables

**Figure 1:**
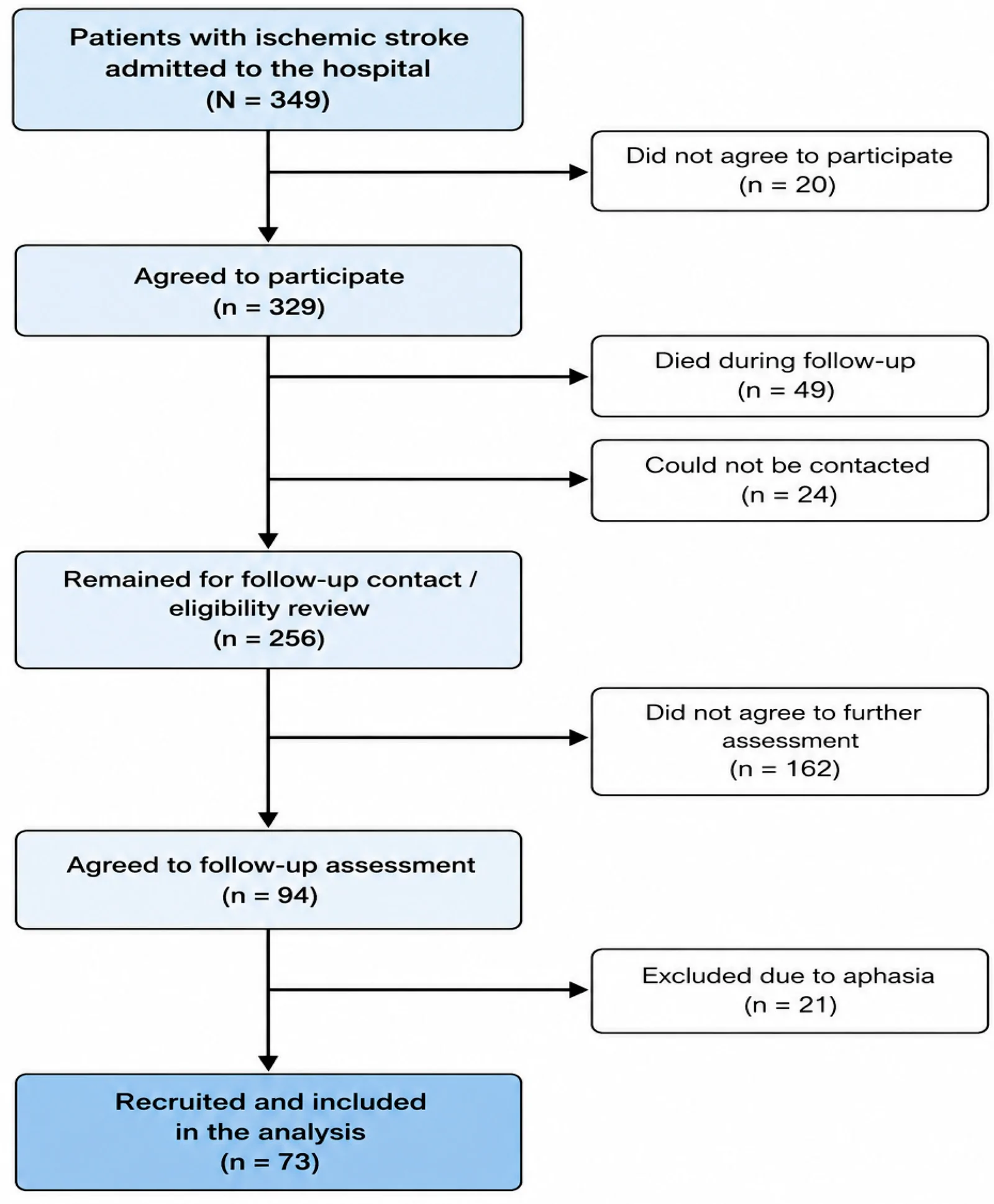
Participant recruitment and selection flow diagram.

**Figure 2:**
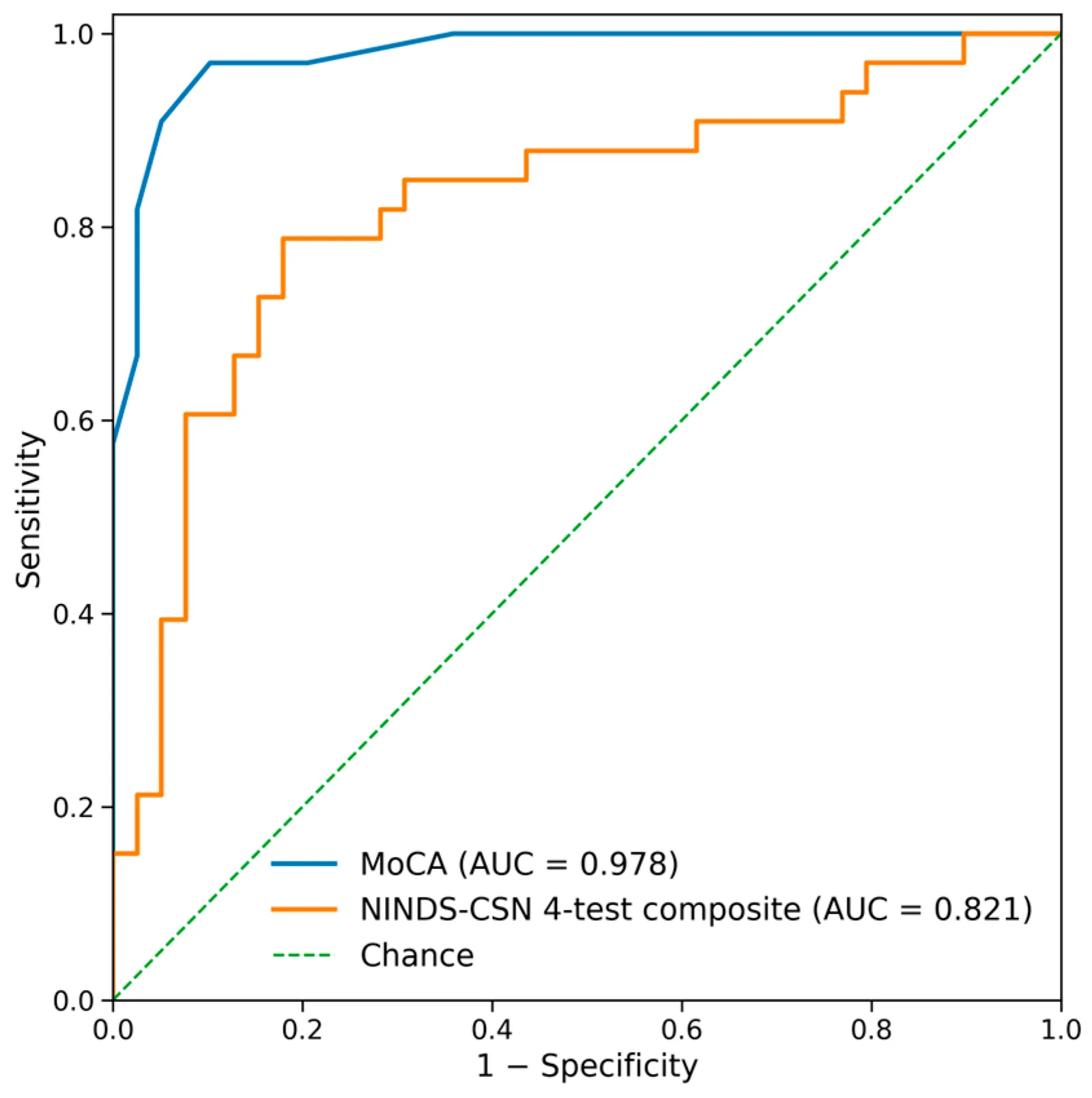
Receiver operating characteristic (ROC) curves comparing the diagnostic performance of the MoCA and NINDS-CSN 4-test composite for detecting PSCI.

**Figure 3:**
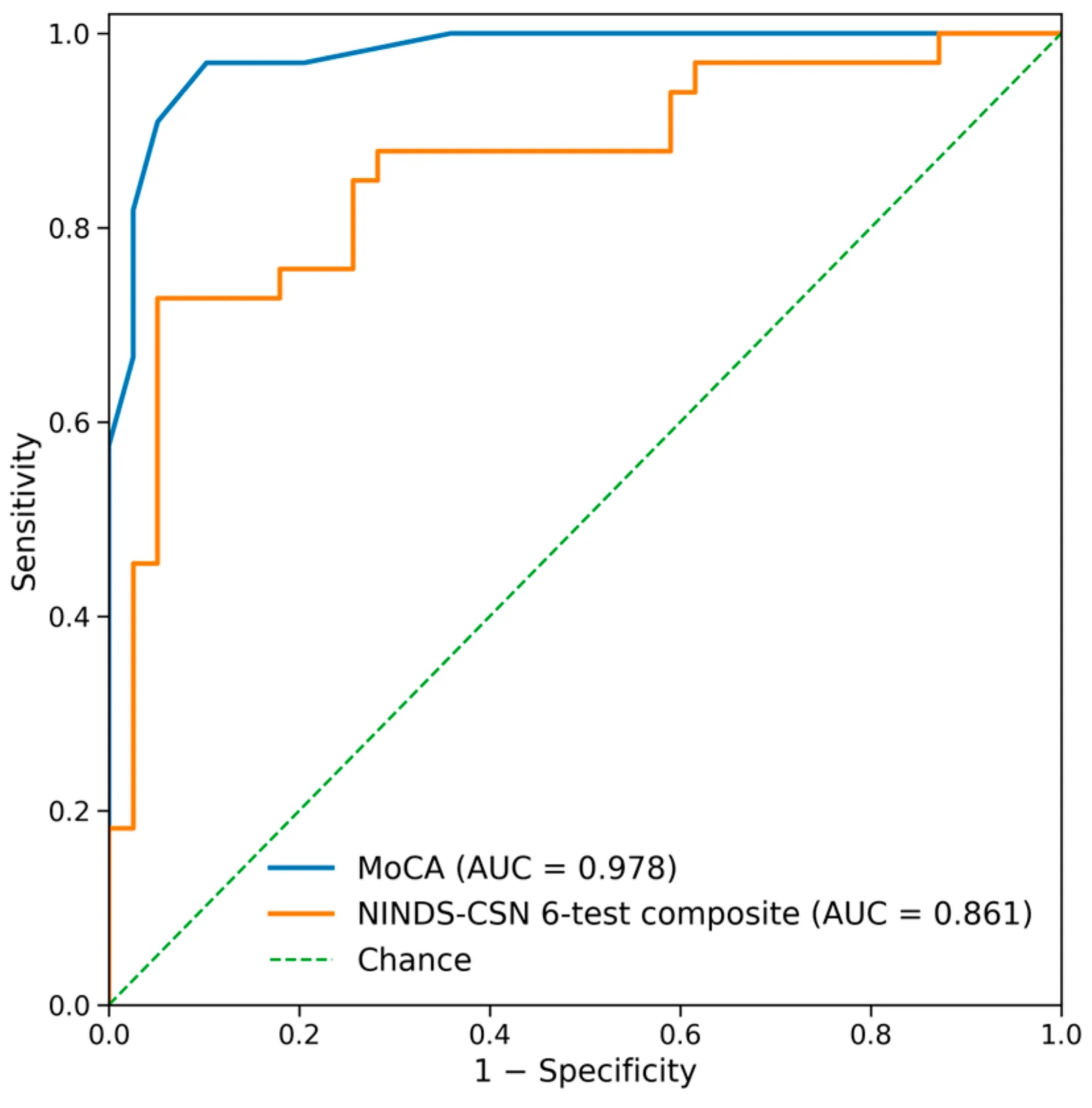
Receiver operating characteristic (ROC) curves comparing the diagnostic performance of the MoCA and NINDS-CSN 6-test composite for detecting PSCI.

**Table 1:** Demographics and neuropsychological battery results in healthy controls versus patients with stroke.

	Controls *N* = 102	Stroke *n* = 73	*p*-Value	Cohen’s d
Age (years)	67.2 ± 6.4	67.5 ± 11.7	0.83	0.032
Female (%)	72.6	47.9	0.002	—
Higher education (%)	51.9	73.9	0.003	—
Urban residence (%)	93.2	83.6	0.02	—
HVLT	24.8 ± 6.1	19.5 ± 7.9	<0.0001	0.767
COWAT F-A-S	33.0 ± 12.2	14.3 ± 15.3	<0.0001	1.377
COWAT—Animals	20.8 ± 5.3	17.0 ± 7.3	<0.0001	0.611
DSST	30.6 ± 10.9	13.0 ± 10.2	<0.0001	1.658
TMT-A	44.8 ± 20.5	79.0 ± 26.0	<0.0001	1.489
TMT-B	89.3 ± 62.2	225.0 ± 99.0	<0.0001	1.703
ADL	6 ± 0	4.9 ± 1.9	<0.0001	0.819
CES-D	6.64 ± 8.18	11.7 ± 9.5	0.0023	0.571
IADL	7.91 ± 0.69	5.9 ± 2.9	<0.0001	0.954

Abbreviations: HVLT—Hopkins Verbal Learning Test; COWAT—Controlled Oral Word Association F-A-S test; COWAT Animals—Controlled Oral Word Association Animals; DSST—Digit Symbol Substitution Test; TMT-A and B—Trail Making Test A and B; ADL—Activities of Daily Living; CES-D Centre for Epidemiologic Studies-Depression Scale; IADL—Instrumental Activities of Daily Living.

**Table 2:** Clinical and neuroimaging characteristics of the stroke cohort by clinical reference diagnosis of PSCI.

	All Stroke Patients (*N* = 73)	Clinical PSCI+ (*n* = 34)	Clinical PSCI− (*n* = 39)	*p*-Value
**Age, years**	67.45 ± 11.71	70.62 ± 11.36	64.69 ± 11.45	0.030
**Female, *n* (%)**	35 (47.9%)	17 (50.0%)	18 (46.2%)	0.743
**Higher education, *n* (%)**	53 (72.6%)	23 (67.6%)	30 (76.9%)	0.375
**Urban residence, *n* (%)**	61 (83.6%)	29 (85.3%)	32 (82.1%)	0.709
**Alcohol use, *n* (%)**	2 (2.7%)	1 (2.9%)	1 (2.6%)	1.000
**Current smoking, *n* (%)**	13 (17.8%)	4 (11.8%)	9 (23.1%)	0.208
**Hypertension, *n* (%)**	63 (86.3%)	30 (88.2%)	33 (84.6%)	0.742
**Type 2 diabetes, *n* (%)**	18 (24.7%)	11 (32.4%)	7 (17.9%)	0.154
**Hyperlipidemia, *n* (%)**	17 (23.3%)	7 (20.6%)	10 (25.6%)	0.610
**Previous ischemic stroke, *n* (%)**	15 (20.5%)	8 (23.5%)	7 (17.9%)	0.556
**Previous TIA, *n* (%)**	14 (19.2%)	9 (26.5%)	5 (12.8%)	0.139
**Atrial fibrillation, *n* (%)**	13 (17.8%)	6 (17.6%)	7 (17.9%)	0.973
**NIHSS < 5, *n* (%)**	23 (31.5%)	8 (23.5%)	15 (38.5%)	0.275
**NIHSS 5–15, *n* (%)**	44 (60.3%)	24 (70.6%)	20 (51.3%)	—
**NIHSS > 15, *n* (%)**	6 (8.2%)	2 (5.9%)	4 (10.3%)	—
**mRS at discharge**	2.29 ± 1.47	2.09 ± 1.48	2.46 ± 1.45	0.256
**Left hemisphere** **infarct, *n* (%)**	28 (38.4%)	17 (50.0%)	11 (28.2%)	0.056
**Right hemisphere** **infarct, *n* (%)**	32 (43.8%)	15 (44.1%)	17 (43.6%)	0.964
**Brainstem/cerebellum infarct, *n* (%)**	19 (26.0%)	7 (20.6%)	12 (30.8%)	0.323
**Basal ganglia infarct,** ***n* (%)**	9 (12.3%)	4 (11.8%)	5 (12.8%)	1.000
**Thalamic infarct, *n* (%)**	5 (6.8%)	5 (14.7%)	0 (0.0%)	0.019
**Moderate-to-severe white-matter disease,** ***n* (%)**	25 (34.2%)	16 (47.1%)	9 (23.1%)	0.031
**Cardioembolic etiology, *n* (%)**	16 (21.9%)	6 (17.6%)	10 (25.6%)	0.410
**Large-artery disease,** ***n* (%)**	12 (16.4%)	5 (14.7%)	7 (17.9%)	0.709
**Small-vessel disease,** ***n* (%)**	31 (42.5%)	12 (35.3%)	19 (48.7%)	0.247
**Other etiology, *n* (%)**	1 (1.4%)	1 (2.9%)	0 (0.0%)	0.466
**Cryptogenic etiology,** ***n* (%)**	13 (17.8%)	10 (29.4%)	3 (7.7%)	0.016

**Table 3:** Neuropsychological and functional results by clinical reference diagnosis of PSCI.

	Clinical PSCI+ (*n* = 34)	Clinical PSCI− (*n* = 39)	*p*-Value	Cohen’s d
**HVLT**	14.82 ± 7.33	23.67 ± 5.77	<0.001	1.352
**COWAT F-A-S**	16.06 ± 9.67	30.51 ± 11.13	<0.001	1.380
**Animal Fluency**	13.29 ± 7.35	19.44 ± 5.92	<0.001	0.928
**DSST**	7.56 ± 7.77	18.51 ± 9.41	<0.001	1.261
**TMT-A, s**	91.53 ± 18.88	67.33 ± 26.13	<0.001	1.050
**TMT-B, s**	273.59 ± 68.81	168.05 ± 93.20	<0.001	1.275
**MoCA**	14.71 ± 5.64	24.44 ± 2.37	<0.001	2.307
**MMSE**	20.38 ± 5.77	28.69 ± 1.96	<0.001	1.983
**ADL**	4.38 ± 2.24	5.49 ± 1.25	0.006	0.620
**CES-D**	15.44 ± 9.87	8.42 ± 7.83	0.003	0.793
**IADL**	4.59 ± 3.39	7.13 ± 1.88	<0.001	0.945

## Data Availability

The data supporting the findings of this study are available from the corresponding authors on request.
